# The Role of Liquid Biopsy in Early Diagnosis of Lung Cancer

**DOI:** 10.3389/fonc.2021.634316

**Published:** 2021-04-16

**Authors:** Cláudia Freitas, Catarina Sousa, Francisco Machado, Mariana Serino, Vanessa Santos, Natália Cruz-Martins, Armando Teixeira, António Cunha, Tania Pereira, Hélder P. Oliveira, José Luís Costa, Venceslau Hespanhol

**Affiliations:** ^1^ Department of Pulmonology, Centro Hospitalar e Universitário São João, Porto, Portugal; ^2^ Faculty of Medicine, University of Porto, Porto, Portugal; ^3^ Laboratory of Neuropsychophysiology, Faculty of Psychology and Education Sciences, University of Porto, Porto, Portugal; ^4^ Institute for Research and Innovation in Health (i3S), University of Porto, Porto, Portugal; ^5^ Institute for Biomedical Sciences Abel Salazar (ICBAS), University of Porto, Porto, Portugal; ^6^ Faculty of Engineering, University of Porto, Porto, Portugal; ^7^ Institute for Systems and Computer Engineering, Technology and Science (INESC TEC), Porto, Portugal; ^8^ Department of Engineering, University of Trás-os-Montes and Alto Douro, Vila Real, Portugal; ^9^ Faculty of Sciences, University of Porto, Porto, Portugal; ^10^ Institute of Molecular Pathology and Immunology of the University of Porto (IPATIMUP), Porto, Portugal

**Keywords:** lung cancer, clinical biomarkers detection, liquid biopsy, cell-free DNA, exosomes, tumor-educated platelets, circulating tumor associated cells

## Abstract

Liquid biopsy is an emerging technology with a potential role in the screening and early detection of lung cancer. Several liquid biopsy-derived biomarkers have been identified and are currently under ongoing investigation. In this article, we review the available data on the use of circulating biomarkers for the early detection of lung cancer, focusing on the circulating tumor cells, circulating cell-free DNA, circulating micro-RNAs, tumor-derived exosomes, and tumor-educated platelets, providing an overview of future potential applicability in the clinical practice. While several biomarkers have shown exciting results, diagnostic performance and clinical applicability is still limited. The combination of different biomarkers, as well as their combination with other diagnostic tools show great promise, although further research is still required to define and validate the role of liquid biopsies in clinical practice.

## Introduction

Lung cancer (LC) is the most common type of cancer and the leading cause of cancer-related mortality worldwide (1). The prognosis is closely related to the stage at diagnosis, with most cases being diagnosed at locally advanced and advanced stages, when curative treatment is no longer possible (2, 3). Thus, to achieve the LC curative treatment, improving overall survival and to diminish the healthcare costs and adverse events related to systemic therapies, the development of novel diagnostic methods that improve the early diagnosis accuracy are of huge importance. Liquid biopsy is a non-invasive, easy and accessible tool for tumor cells or tumor-derived products detection in body fluids, with the potential of overcome the limitations of the strategies currently used for LC early detection. Indeed, the molecular assessment of tumor-derived components from peripheral blood is of high clinical value, besides to represent promising clinical biomarkers (4). In this sense, and given the above highlighted aspects, this review provides an overview on the utility of liquid biopsy components as early diagnostic biomarkers.

## Liquid Biopsy in Early Diagnosis: What is the Rationale?

Thoracic imaging is the traditional method used for early detection of LC, that occur either as an incidental finding or integrated in a screening program. The National Lung Screening Trial (NLST) showed a reduction of 20% in LC specific mortality rate with chest low dose computed tomography (LDCT) screening among high risk individuals, when compared with chest X-ray (5). Recently, the same trial with a median follow-up of 12 years confirmed consistent benefits in terms of LC-related deaths reduction (6). However, the rate of false positives, overdiagnosis and unnecessary invasive procedures still remain major concerns (7).

Tissue biopsies are essential for LC diagnosis. Despite imaging-guided percutaneous needle biopsy has been considered as a relatively safe procedure for peripheral lesions diagnosis, it is not free of complications (8, 9). Bronchoscopy has also a pivotal role in LC diagnosis, with flexible bronchoscopy being the more useful test for central lesions, whereas navigational bronchoscopy and radial endobronchial ultrasound (EBUS) display higher sensitivities for peripheral lesions (10). Nonetheless, although uncommon, complications may occur (11). Tissue biopsies, although of extreme interest and usefulness, have also limitations. For instance, due to tumor heterogeneity, a single biopsy may not be representative of the entire tumor and may misjudge the complexity of its genetic aberrations (12). Also, the primary tumor and its metastases may have significant inter- and even intra-tumor heterogeneity (12). Thus, the lack of enough tissue sample to carry out a complete tumor characterization, comprising histology, immunohistochemistry and genetic analysis, essential for therapeutic decision and prognosis definition, often represents an issue in clinical practice. Although transthoracic needle aspiration or biopsies perform better than bronchoscopic procedures in peripherical lung lesions diagnosis, this technique only provides the confirmation of diagnosis in 90% of LC cases, with 20–30% false negatives (10).

More recently, other strategies have been explored, with circulating biomarkers being target of an extreme attention and interest. Briefly, biomarker is defined as a feature that can be objectively measured and evaluated as an indicator of biological and pathogenic processes, or pharmacologic responses to therapeutic intervention (13). Circulating or other body fluid, especially respiratory samples biomarkers may be viewed as key strategies for improving LC early diagnosis. In this way, liquid biopsy, as a non-invasive, safe and easy procedure, has the potential to improve the currently used strategies for LC diagnosis, either in screening setting or as an alternative diagnostic tool, either alone or as complementary data for imaging findings. Several clinical applications have been reported in LC, including patients stratification, therapeutic decision, and disease monitoring either after surgery or during systemic therapies, enabling to detect the acquired resistance (14). Although the role of liquid biopsy in LC early detection is not yet defined, there is increasing evidence about its potential applications.

## The Biology Behind Liquid Biopsies

**Figure 1:**
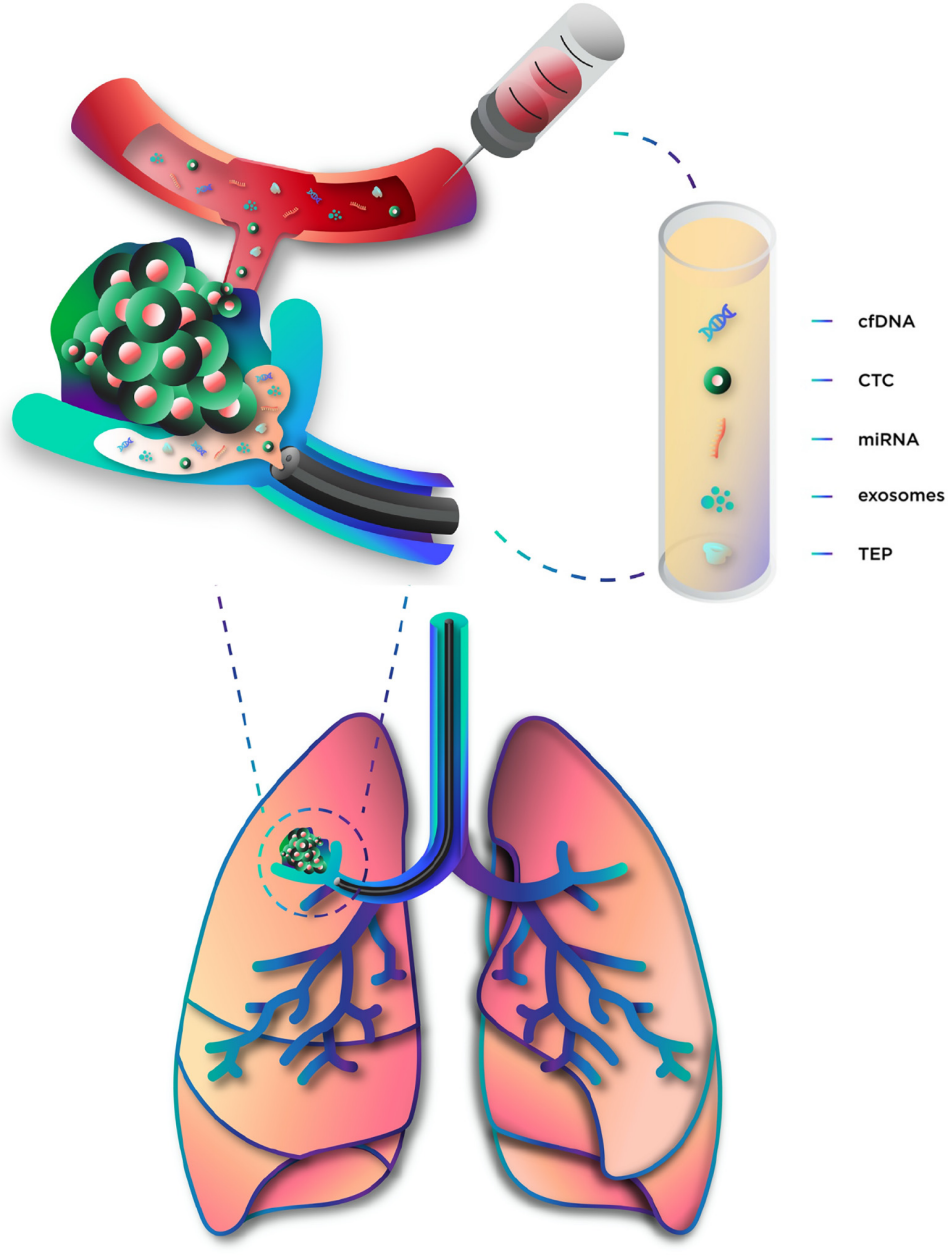
Components of liquid biopsy. cfDNA, circulating cell-free DNA; CTC, circulating tumor cells; miRNA, microRNA; TEP, tumor-educated platelets.

Circulating tumor cells (CTCs) and circulating cell-free DNA (cfDNA) are the most studied liquid biopsy-derived biomarkers, but many others have also been investigated (15) **(** **Figure 1** **).** **Table 1** summarizes their advantages and limitations.

**Table 1:** Summary of advantages and limitations in LC diagnosis according to liquid biopsy-based biomarker.

Biomarkers	Advantages	Limitations	References
cfDNA	Increased in cancer patients comparing to healthy individuals; Genetic and epigenetic alterations reflect those of the original tumor; Representation of tumor heterogeneity and dynamics; Highly sensitive assays available (PCR, NGS).	Markedly diluted compared to germline circulating DNA; Positively correlates with tumor size and staging; Increased in some benign or premalignant conditions; High costs.	(16, 17–18)
CTC	Allows morphologic analysis and tumor molecular characterization; Correlates with prognosis; Emerging enrichment and characterization techniques.	No validated assay; Rare in bloodstream and surrounded by blood cells; Epithelial to mesenchymal transition with loss of epithelial-specific markers; Role in cancer spreading still to be clarified.	(17, 19, 20)
miRNA	Different profiles among early-stage cancer patients; Stable in most types of body fluids (e.g. respiratory samples); Released by several structures (e.g. exosome, TEPs). Commercial kits available for collection.	High variability, according to patients and technologies, requiring normalization methods; Quantification and detection methods need to be validated Unspecific for a cancer type.	(21–22)
Exosomes	Contains several types of biomarkers such as proteins and nucleic acids; Increased in lung cancer patients; Stable and accessible in most types of body fluids; Commercial kits available for isolation.	The extraction approach, detection and characterization methods are challenge and require standardization; High costs	(23–24)
TEP	Platelet mRNA profile is distinct in cancer patients; Abundant; Easily isolated; Acquire specific RNA from tumor cells reflecting its genetic alterations; Dynamic mRNA repertoire due to short life-span.	No validated assay nor standardized approach; Reproducibility; Detection techniques not widely available; Time consuming and requires extensive computational resources.	(25–26)

### Circulating Cell-Free DNA

Tumor cells release DNA fragments into the bloodstream or other anatomic-related body fluid, such as urine or pleural fluid. It is known that cancer patients have higher levels of cfDNA than healthy individuals and, since the tumor volume correlates with cell turnover and death, circulating tumor DNA (ctDNA) concentration increases with tumor size (15, 27). Probably, most fragments result from apoptosis, as they range from 180 to 200 base pairs (16). In addition, smaller and larger fragments have also been reported, suggesting that necrosis is also a probable source (28). Macrophages seem to contribute to the releasing process after necrotic tumor cells phagocytosis (29). When in circulation, cfDNA can be linked to proteins or, alternatively, be transported by vesicles, such as exosomes or apoptotic bodies, through a process that, although not completely clarified, seems to contribute to distant spreading and metastasis (30, 31). In addition, there is evidence that a fraction of tumor DNA circulates in the blood linked to the blood cells surface (i.e. erythrocytes and leukocytes) (31, 32). The genetic alterations of cfDNA reflect the genomic alterations of the original tumors and include point mutations, rearrangements, amplifications and gene copy variations (15).

### Circulating Tumor Cells

CTCs released by the primary tumor can be detected in the bloodstream and represent not only an attractive diagnostic method, as a morphologic analysis can be performed, but also an opportunity for molecular characterization, since DNA, RNA and protein information can be obtained (15). During the metastatic process, the tumor cells separate from the primary tumor, migrate through the surrounding tissue and reach lymphatic or blood circulation (33). Two different ways of tumor cell migration have been proposed (34). First, active migration implies that a single or a cluster of tumor cells has gained the ability to move through the extracellular matrix and basement membranes (35). Second, passive migration refers to the growth of tumor mass that pushes single or clusters of tumor cells into the circulation (36) and, as this process is common in epithelial malignancies, CTC frequently maintains the epithelial phenotype and presents epithelial-specific markers, such as the epithelial cell adhesion molecule (EpCAM) (33, 34). Epithelial malignancies may also shift their phenotype from epithelial to mesenchymal. Although the meaning of the transition is still on debate, an association with the ability of becoming invasive has been suggested (37, 38). In these cells, EpCAM is downregulated and, thus, cannot be detected by conventional EpCAM-based methods (33). Surviving in the bloodstream is not easy for CTC, since many barriers need to be overcome, namely the forces and stresses created by the blood flow, anoikis and the immune system (33). During this phase, CTCs can be detected in the bloodstream and serve as a biomarker. Tamminga et al. (39) studied the release of CTCs during surgery and identified higher CTCs counts by CellSearch system in the pulmonary vein compared to peripherical circulation, suggesting a clearance mechanism. Since two groups of cells were detected in pulmonary vein samples—the real CTC and benign epithelial cells, the difference between peripherical and central circulation may be explained by the lack of survival ability of benign epithelial cells due to lower tolerance of shearing forces and the mesenchymal environment, leading to their fast clearance or destruction (39). Still, CTCs presence in pulmonary vein at time of surgery was found to be an independent predictor of LC-specific relapse and their genomic features greatly overlap with those of the metastasis detected 10 months later (19). In fact, extravasion from vessels takes place when blood flow slows down, allowing the CTCs to attach the endothelium (40). Once in the metastatic site, tumor cells can initiate a quiescent state, called cancer dormancy (41), until the new surrounding microenvironment allows proliferation (33).

### MicroRNA

In opposition to free RNA molecules that generally do not persist in circulation, cell-free miRNAs can be detected in blood of cancer patients. These fragments of single-stranded non-coding RNA, with a length of 19 to 25 nucleotides, play an important role in gene expression regulation. Mature miRNAs may present in a complex called multiprotein RNA-induced silencing complex (miRISC), which regulates gene expression at a translational level by targeting messenger RNAs (mRNAs) (42). A single miRNA can act on a large number of target mRNAs (43), and a single mRNA target may have multiple miRNA binding sites as well (44), allowing complex combinatorial gene regulation mechanisms. These molecules are involved in several biological processes, such as cell development, differentiation, apoptosis and proliferation (45), and, therefore, changes in the normal cellular miRNA profile can lead to functional abnormalities. In fact, loss or amplification of miRNA genes have been reported in a variety of cancers (46). Since some of the miRNA targets are oncogenes and tumor-suppressor genes, abnormal miRNA levels may result in oncogene activation and/or loss of tumor suppressing mechanisms, which eventually lead to cancer (42). Briefly, distinct miRNAs profiles on tissue and fluid samples seem to discriminate between healthy and tumor tissues (47). MiRNAs are released into the blood stream and surrounding tissues through exosomes, apoptotic bodies, protein–miRNA complexes, and tumor-educated platelets (TEP) (21), which, in conjunction with their remarkable stability (48) makes miRNA profiling a promising tool for cancer detection.

### Exosomes

Exosomes are extracellular vesicles, with a diameter of 40–100 nm, derived from the progressive accumulation of intraluminal vesicles that are released into the extracellular space by fusion with plasma membrane (49–51). Its content, such as nucleic acids and proteins, and function are intrinsically related to the cells of origin. Tumor cells are known to release greater amounts of exosomes than healthy cells and these structures can be found in almost all body fluids (23, 51, 52). This vesicles mediate cell-to-cell communication and affect many biological processes in LC, contributing to its progression, angiogenesis and metastasis (53, 54). Several possible mechanisms through which exosomes communicate with target cells have been described. Exosomal membrane proteins can interact directly with the receptors of a target cell and activate intracellular signaling. Additionally, exosomes can merge with the target cell membrane and release its contents into the target cell. This content, that can include proteins, mRNAs, miRNAs and DNA can promote a multiplicity of signaling events in the target cell (49, 54). In fact, the exosome and its molecular content represent a source of exclusive information on tumor cell.

### Tumor-Educated Platelets

Platelets are anucleate cells originating from megakaryocytes in bone marrow, known for their role in hemostasis and thrombosis. Despite that, platelets have emerged as having a major impact in both progression and spreading of several solid tumors, including LC (55, 56). Tumor growth, progression and spreading require specific changes in tumor cells and in the surrounding microenvironment, being many of them similar to the physiological role of platelets (55). Although their exact role in cancer is still under investigation, several hypotheses have been proposed. First, TEPs have the ability to create a favorable tumor microenvironment supporting the proliferative signals release, promoting tumor progression, metastasis and angiogenesis in LC (57). Second, TEPs prevent immune destruction by forming a layer that protects the circulating tumor cells from natural killer, other immune cells and from shear forces of circulatory system (58). This coating mechanism may lead to MHC class I transfer to the tumor cells surface, making them unrecognizable by immune cells (59, 60) and contributing to distant metastasis formation (61, 62). Third, TEPs promote invasion and metastasis through releasing several growth and proangiogenic factors, such as platelet-derived growth factor (PDGF) and transforming growth factor *β* (TGF-β) (63). Fourth, TEPs seems to induce angiogenesis by delivering proangiogenic factors to the tumor and stimulating the expression of its own angiogenic factors, such as vascular endothelial growth factor (VEGF), platelet-derived growth factor (PDGF) and basic fibroblast growth factor (bFGF) (64, 65). Lastly, TEPs directly interact with tumor cells by acquiring biomolecules, as well as indirectly in response to external signals (55, 66).

## Liquid Biopsy Components as Early Detection Biomarkers: Current Evidence

### Circulating Cell-Free DNA

CfDNA has been extensively studied in LC and its concentration was found to be increased in LC patients (67). However, some important potential limitations have been discussed concerning its utility as an early detection biomarker. First, cfDNA is markedly diluted compared to circulating germline DNA (68) complicating the detection process. Second, it has been estimated that a minimum 10 cm^3^ of tumor volume is required to quantify variant allele frequencies of 0.1%, thus hindering early stage tumor detection (69). Definitely cfDNA concentration correlates with tumor size and staging (17), being early-stage LC patients less prone to have representative samples than patients at an advanced stage. Abbosh and colleagues identified several factors related to cfDNA detection, including non-adenocarcinoma histology, high Ki67 expression and lymphovascular invasion. Also, PET FDG avidity was shown to predict cfDNA detection (69). Third, healthy individuals frequently have free DNA in circulation, though in smaller concentrations (70), and benign conditions, such as infections, cardiovascular diseases or other lung diseases are associated to increased cfDNA levels (71–73). However, these limitations may be overcome using highly sensitive genotyping assays, such as digital polymerase chain reaction (PCR) and next-generation sequencing (NGS). Also, a size-based pre-selection of DNA fragments could improve both sensitivity and specificity (74). While PCR methods can only target specific sites in a pre-defined gene and are not able to detect complex genomic alterations, such as gene fusions, NGS-based assays are multiplex methods, also known as massively parallel sequencing assays, allowing a concurrent detection of somatic mutations, including single-nucleotide and copy number variations, gene insertions, deletions or fusions. However, as the portion of sequencing genome increases, a loss of coverage is observed, limiting the ability to call a variant with confidence (75). Thus, the use of panels of primers or probes targeting hotspots or exons of pre-selected genes, such as hybrid capture NGS (76) or amplicon-based NGS (77, 78) is a reasonable strategy for cfDNA detection (79). The correct interpretation of cfDNA genotyping can be challenging, with the major limitation being the rate of false negatives that may result from the assay technical limits or, most importantly, from the cfDNA concentration, especially in early stages (18). On the other side, false positives may also occur due to sequencing errors or by the presence of other tumor or premalignant condition (e.g. in clonal hematopoiesis) (79). One must be aware that considering the primary tumor as reference may lead to misclassification as false positives, since a genetic alteration may be present in a tumor site and absent in other due to tumor heterogeneity (79).

Several studies have addressed the potential of cfDNA for early detection, either focusing in its concentration (**Table 2**) or genetic (**Table 3**) and epigenetic alterations, more specifically methylation patterns (**Table 4**).

**Table 2:** cfDNA plasma concentration performance as a biomarker for lung cancer diagnosis.

Study	Year	Assay	Study population	Diagnostic performance			
				Cut-off	S	E	AUC
Sozzi (80)	2001	DNA DipStick TM Kit (Invitrogen, Carlsbad, CA)	43 healthy controls 84 patients with radically resected NSCLC (47 ADCs, 25 SCC and 12 others)	6–25 ng/ml	75%	86%	0.844
			Stages: IA: 14; IB: 32; II: 15; III: 23				
Sozzi (81)	2003	RT-PCR using hTERT	100 controls 100 consecutive patients with NSCLC (58 ADC, 34 SCC, 8 others)	25 ng/ml	46%	99%	0.940
			Stages: IA: 16; IB: 18; IIB: 25; IIIA: 33; IIIB: 5; IV: 3				
Gautschi (82)	2004	RT-PCR	46 healthy controls 185 NSCLC patients (81 ADC, 49 SCC, 37 LCC and 18 undifferentiated)	10 ng/ml	–	98%	–
			Stages: I-II: 19; III: 62; IV: 104				
Herrera (83)	2005	RT-PCR using human β-actin gene	11 healthy volunteers; 38 esophageal cancer; 28 GERD 25 NSLC patients undergoing surgery	14.0 μg/L	48%	100%	0.630
			Stages: I: 10; II: 4; III: 3; IV: 1; Unknown: 7				
Ludovini (84)	2008	RT-PCR	66 controls 76 consecutive patients with NSCLC undergoing surgery (37 SCC, 28 ADC and 11 LCC)	3.25 ng/ml	80%	61%	0.820
			Stages: I: 20; II: 40; IIIA: 11; IIIB: 5				
Szpechcinski (85)	2015	RT-PCR using human β-actin	40 healthy volunteers 101 patients with chronic respiratory inflammation (34 COPD, 35 sarcoidosis, 32 asthma) 50 resectable NSCLC patients (24 ADC, 22 SCC and 4 others)	2.80 ng/ml	90%	81%	0.900
			Stages: I: 22; II: 20; IIIA: 8				
Szpechcinski (86)	2016	RT-PCR using human β-actin	16 healthy controls 28 subjects with benign lung tumors 65 NSCLC patients (28 ADC, 27 SCC, 10 others)	2.80 ng/ml	86%	61%	0.800
			Stages: I: 30; II: 23, III: 12				
Sozzi (87)	2009	RT-PCR using hTERT	1035 subjects included in a CT screening program (annually CT). During the 5-year follow-up period, 956 remained cancer free, 38 developed LC, and 41 developed other tumors	–	–	–	0.496
Paci (88)	2009	RT-PCR using hTERT	79 healthy controls. 151 NSCLC patients (65 SCCl, 61 ADC, 12 bronchioloalveolar, 3 LCC, 2 typical carcinoid, 8 others)	2 ng/ml	86%	47%	0.790
			Stages: IS: 1; IA: 33; IB: 44; IIA: 5; IIB: 12; IIIA: 24; IIIB: 18; IV: 4				
Yoon (89)	2009	RT-PCR using human β-actin gene	105 healthy controls 102 LC patients (67 ADC, 16 SCC, 10 SCLC and 9 others)	–	–	–	0.860
			Stages in SCLC: localized: 5; extensive: 4 Stages in NSCLC: I: 8; II: 2; III: 19; IV: 64				
Van der Drift (90)	2010	RT-PCR using human β-actin gene	21 controls 46 untreated NSCLC patients (21 SCC, 20 ADC, 5 LCC)	>32 ng/ml	52%	67%	0.660
			Stages: I: 11; II: 6; III: 12; IV: 15; Unknown: 2				
Catarino (91)	2012	RT-PCR using hTERT	205 controls 104 NSCLC patients (38 SCC, 54 ADC and 12 others)	20 ng/ml	79%	83%	0.880
			Stages: I/II: 4; III/IV: 100				

ADC, adenocarcinoma; AUC, area under the curve; E, specificity; hTERT, human telomerase reverse transcriptase gene; IS, in situ; LC, lung cancer; LCC, large cell carcinoma; NSCLC, non-small cell lung cancer; S, sensitivity; SCC, squamous cell carcinoma; RT-PCR, real time polymerase chain reaction.

**Table 3:** Plasma cfDNA genetic alterations performance as biomarker for lung cancer diagnosis.

Study	Year	Assay	Genetic alteration	Study population	Diagnostic performance		
					S	E	AUC
**Single biomarker**							
Zhao (92)	2013	Mutant-enriched PCR and sequencing	EGFR mutations (exon 19 and 20) and EGFR exon 19 deletions	111 NSCLC patients including 35 SCC, 73 ADC and 3 others. Stages: I: 22; II: 10; IIIA: 19; IIIB: 14; IV: 46	36%	96%	–
Jing (93)	2014	HRM analysis	EGFR mutations (exons 18, 19, 20 and 21)	120 NSCLC patients including 70 ADC and 50 non-ADC. Stages: I/II: 38; III/IV: 82	78%	97%	–
Uchida (94)	2015	NGS	EGFR mutations (exon 19, 20, 21)	288 NSCLC patients including 274 ADC, 7 SCC, and 7 others Stages: I: 64; II: 19; III: 53; IV: 146	Exon 19 deletions: 51% L858R mutation: 52%	Exon 19 deletions: 98% L858R mutation: 94%	–
Fernandez-Cuesta (95)	2016	NGS	TP53 (exons 2 to 10)	123 non-cancer controls; 51 SCLC patients Stages: I: 7; II: 7; III: 28; IV: 9	49%	89%	–
Wan (96)	2018	ARMS-PCR	EGFR mutations (exon 19 deletion, T790M, L858R)	69 controls; 284 early-stage NSCLC patients (35 ADC, 231 SCC and 18 others) Stages: I: 107; II: 177	14%	92%	–
Wei (97)	2018	EFIRM	EGFR mutations (exon 19 deletion and L858R)	23 patients with benign pulmonary nodules21 early-stage ADC patients (12 L858R and 9 exon19 deletion EGFR variants) Stages: I: 18; II: 3	Exon 19 deletions: 77% L858R mutation: 92%	95%	Exon 19 deletions: 0.978 L858R mutation: 0.973
**Combination biomarker**							
Newman (98)	2014	CAPP-Seq	139 cancer-related genes	5 healthy controls17 NSCLC patients (14 ADC, 2 SCC and 1 LCC) Stages: I: 4; II: 1; III: 6; IV: 6	85%	96%	0.950
Guo (99)	2016	NGS	50 cancer-related genes	41 NSCLC patients (33 ADC, 6 SCC, 2 others) Stages: I: 23; II: 7; III: 10; IV: 1	69%	93%	–
Chen (100)	2016	NGS	50 cancer-related genes	58 NSCLC patients (51 ADC and seven SCC) Stages: I: 46; II: 12	54%	47%	–
Cohen (101)	2018	CancerSEEK (NGS and protein immunoassay)	8 proteins and 16 cancer-related genes	812 healthy controls1005 patients with stage I to III cancers including 103 NSCLC and 1 SCLC Stages: I: 46; II: 21; III: 31	70%	99%	0.910
Ye (102)	2018	NGS	140 cancer-related genes	35 lung surgery candidate nodule patients (four benign nodule patients, 31 LC patients: 2 ADC IS, 25 ADC, 1 SCC, 3 other) Stages: I: 21; II: 5; III: 4; not defined: 1	33%	100%	–
Peng (103)	2019	NGS	65 cancer-related genes	56 benign lung lesions patients136 LC patients (100 ADC, 28 SCC, 1 SCLC, 7 others) Stages: I: 87; II: 29; III: 17; IV: 3	69%	96%	–
Tailor (104)	2019	NGS		16 benign lung lesion patients17 LC patients (10 ADC, 6 SCC, 1 LCC) Stages: I: 8; II: 2; III: 5; IV: 2	82%	–	–
Leung (105)	2020	COLD–PCR assay coupled with high-resolution melt analysis	KRAS, EGFR, and TP53	26 controls192 patients referred to surgery (106 primary LC, 54 secondary cancer, 6 another primary thoracic malignancy) Stages: I: 52; II: 33; III: 16; IV: 1; Missing: 4	75%	89%	–

ADC, adenocarcinoma; ARMS-PCR, Amplification-refractory-mutation system-based PCR assays; AUC, area under the curve; CAPP-Seq, CAncer Personalized Profiling by deep Sequencing; COLD, Lower denaturation temperature; E, specificity; EFIRM, Electric field-induced release and measurement; HRM, High resolution melting; IS, in situ; LC, lung cancer; LCC, large cell carcinoma; NSCLC, non-small cell lung cancer; S, sensitivity; SCC, squamous cell carcinoma; SCLC, small cell lung cancer.

**Table 4:** cfDNA hypermethylation performance as biomarker for lung cancer diagnosis.

Study	Year	Assay	Methylated genes	Study population	Sample	Diagnostic performance		
						S	E	AUC
**Single-Dual biomarker**								
Wang (106)	2007	Methylation-Specific RT-PCR	RASSF1A	15 healthy controls 35 benign pulmonary diseases patients 80 LC patients (40 ADC, 26 SCC, nine adenosquamous carcinoma, five SCLC) Stages: I: 9; II: 18; III: 29; IV: 24	Serum	34%	100%	–
Schmidt (107)	2010	Methylation-Specific RT-PCR	SHOX2	242 controls 281 LC patients (109 ADC, 103 SCC, 37 NOS NSCLC, 29 SCLC, three others) Stages: I: 59; II: 43; III: 108; IV: 62; unknown: 9	Bronchial aspirates	68%	95%	0.860
Kneip (108)	2011	Methylation-Specific RT-PCR	SHOX2	155 controls 188 LC patients (38 SCC, 31 SCC, 15 SCLC, 104 other) Stages: I:37; II:29; III:53; IV:42; unknown:27	Plasma	60%	90%	0.780
Hwang (109)	2011	Pyrosequencing	HOXA9	51 healthy controls 58 benign lung diseases patients 76 LC patients (42 ADC and 34 SCC). Stages: I: 14; II: 5; III: 28; IV: 29	Induced sputum	71%	55%	0.969
Dietrich (110)	2012	Epi proLung BL	SHOX2 and PTGER4	125 controls 125 LC patients (26 ADC, 28 SCC, 40 SCLC, 9 NSCLC NOS, 32 others)	Bronchial aspirates	78%	96%	0.940
Ponomaryova (111)	2013	Methylation-Specific RT-PCR	RARB2and RASSF1A	32 healthy donors 60 NSCLC patients (40 SCC and 20 ADC) Stages: I/II: 20; III: 40	Plasma and cell-surface-bound circulating DNA	85%	75%	–
Powrózek 2014 (112)	2014	Methylation-specific RT-PCR	Septin 9	100 healthy controls 70 LC patients (20 ADC, 20 SCC, 23 SCLC, seven others) Stages: IIA–IIIA: 23; IIIB–IV: 47	Plasma	44%	92%	
Konecny (113)	2016	Epi proLung BL	SHOX2	69 suspected LC patients; 31 excluded LC (controls) and 38 LC confirmed including 28 NSCLC and one SCLC. Stages: I–II: 5; III–IV: 30; unknown: 3	Bronchial lavage	89%	85%	0.890
					Plasma	81%	79%	0.870
Powrózek (114)	2016	Methylation-specific RT-PCR	DCLK1	95 healthy controls 65 LC patients (22 ADC, 20 SCC, 19 SCLC, four others) Stages: IIA–IIB: 7; IIIA: 21; IIIB–IV: 37	Plasma	49%	92%	–
Ren (115)	2017	Methylation-specific RT-PCR	SHOX2 and RASSF1A	130 controls (112 benign lung disease patients and 18 patients with other malignancies) 52 patients with no exact diagnosis 123 LC patients including 82 ADC, 17 SCC, eight SCLC, 16 others Stages: 0: 4; I: 47; II: 13; III: 19; IV: 25; unknown: 15	Bronchoalveolar lavage	72%	90%	–
Nunes (116)	2019	Methylation-specific RT-PCR	4 genes: APC, HOXA9, RARβ2, and RASSF1A	28 benign lung diseases patients 129 LC cancer patients (65 ADC, 42 SCC, 19 SCLC) Stages: I: 15; II: 11; III: 27; IV: 76	Plasma	APC: 25% RASSF1A: 24% APC and RASSF1A: 38%	APC: 96% RASSF1A: 95% APC and RASSF1A: 93%	APC: 0.622 RASSF1A: 0.591
**Combination biomarker**								
Fujiwara (117)	2005	Methylation-Specific RT-PCR	RARβ, p16^INK4a^, DAPK, RASSF1A, and MGMT	100 non-malignant diseases patients nine other malignancies 91 LC patients (64 ADC, 21 SCC, four SCLC, two carcinoid). Stages: I: 53; II: 7; III: 22; IV: 9	Serum	50%	85%	–
Hsu (118)	2007	Methylation-Specific RT-PCR	BLU, CDH13,FHIT, p16, RARβ, and RASSF1A	36 cancer-free controls 63 NSCLC patients (41 ADCs, 13 SCC) Stages: I–II: 41; III–IV: 21; Not staged: 1	Plasma	73%	82%	
Zhang (119)	2011	Methylation-Specific RT-PCR	9 genes: APC, CDH13, DLEC1, EFEMP1, KLK10, p16^INK4A^, RARβ, RASSF1A, SFRP1	50 cancer-free controls 110 NSCLC patients (Stage I/II)	Plasma	90% APC, RASSF1A, CDH13, KLK10 and DLEC1: 84%	58% APC, RASSF1A, CDH13, KLK10 and DLEC1: 74%	–
Begum (120)	2011	Methylation specific RT-PCR	6 genes: APC, AIM1, CDH1, DCC, MGMT and RASSF1A	30 controls 76 LC patients (36 ADC, 26 SCC, 14 others) Stages: I: 41; II: 17; III: 11; IV: 5; unknown: 2	Serum	84%	57%	–
Nikolaidis (121)	2012	Methylation specific RT-PCR	4 genes: TERT, WT1, p16 and RASSF1	109 controls; 139 LC cases (22 ADC, 31 SCC, 39 SCLC, 16 LCC, 31 others) Stages: T1: 46; T2: 91; T3: 20; T4: 53; N0: 94; N1: 35; N2: 63; N3: 13	Bronchial lavage	82%	91%	–
Diaz-Lagares (122)	2016	Pyrosequencing	4 genes: BCAT1, CDO1, TRIM58, and ZNF177	Bronchial aspirates cohort: -29 cancer-free controls -51 LC patients (17 ADC, 19 SCC, 11 NSCLC NOS, 4 other) Stages: I: 5; II: 6; III: 21; IV: 18; unknown: 1 BAL cohort: -29 cancer-free controls -82 LC patients (25 ADC, 40 SCC, 12 SCLC, 5 others) Stages: I: 17; II: 8; III: 20; IV: 18; unknown: 19 Sputum cohort: -26 cancer-free controls -72 LC patients (38 ADC, 24 SCC, 5 SCLC, 4 other) Stages: I: 12; II: 13; III: 23; IV: 19; unknown: 5	Bronchial aspirates	84%	81%	0.910
					Bronchioalveolar lavages	~80%	~80%	0.850
					Sputum	~65%	~65%	0.930
Ma (123)	2016	Quantum dots combined with FRET	PCDHGB6, HOXA9 and RASSF1A	50 controls 50 NSCLC patients (24 ADC and 16 SCC) Stages: I: 23; II: 17	Bronchial brushing	80%	100%	0.907
Hulbert (124)	2017	Methylation-specific RT-PCR	6 genes: SOX17, TAC1, HOXA7, CDO1, HOXA9, ZFP42	60 cancer-free controls 150 LC cases (121 ADC, 26 SCC, 3 others) Stages: IA–IB: 136; IIA: 14	Sputum	TAC1, HOXA17 and SOX17: 93% 6 genes, age, PY, COPD and FVC: 91%	TAC1, HOXA17 and SOX17: 89%	TAC1, HOXA17 and SOX17: 0.890 6 genes, age, PY, COPD and FVC: 0.850
					Plasma	CDO1, TAC1 and SOX17: 86% 6 genes, age, PY, COPD and FVC: 85%	CDO1, TAC1 and SOX17:78%	CDO1, TAC1 and SOX17: 0.770 6 genes, age, PY, COPD and FVC: 0.890
Ooki (125)	2017	Methylation-specific RT-PCR	6 genes: CDO1, HOXA9,AJAP1, PTGDR, UNCX, and MARCH11	42 controls 43 primary NSCLC with matched serum samples from stage IA ADC 40 serum samples from stage IA SCC 70 pleural effusions samples 49 ascites samples	Serum	ADC: 72%SCC: 60%	71%	–
					Pleural effusions	4-gene panel (CDO1, PTGDR, UNCX, and MARCH11): 70% 5-gene panel (CDO1, AJAP1, PTGDR, UNCX, and MARCH11): 76%	4-gene panel (CDO1, PTGDR, UNCX, MARCH11): 85% 5-gene panel (CDO1, AJAP1, PTGDR, UNCX, MARCH11): 76%	–
Hubers (126)	2017	Methylation-specific RT-PCR	7 genes: RASSF1A, APC, cytoglobin,3OST2, PRDM14, FAM19A4 and PHACTR3	219 controls 56 LC patients (34 ADC,7 SCC, 2 SCLC, 13 others Stages: I:36, II:4, III:6, IV:10	Sputum	17%	93%	–
Liang (127)	2019	Methyl-seq	9 genes	27 controls 39 LC patients (32 ADC, 6 SCC and 1 other) Stages: IA: 20; IB: 7; IIA: 1; Later stages: 10; unknown: 1	Plasma	80%	85%	0.820

ADC, adenocarcinoma; AUC, area under the curve; COPD, chronic obstructive pulmonary disease; E, specificity; FRET, fluorescence resonance energy transfer; FVC, forced vital capacity; IS, in situ; LC, lung cancer; LCC, large cell carcinoma; NSCLC, non-small cell lung cancer; NOS-NSCLC, not otherwise specified non-small cell lung cancer; PY, pack-years; S, sensitivity; SCC, squamous cell carcinoma; SCLC, small cell lung cancer.

#### cfDNA Concentration

An early study by Sozzi et al. (80) showed that plasma cfDNA concentration was higher among NSCLC patients, mostly with localized disease, than in healthy controls.

Real-time quantitative polymerase chain reaction (RT-PCR) amplification of the human telomerase reverse transcriptase gene (hTERT) was used as an indicator of global amount of plasma cfDNA in several studies (81, 87, 88, 91). The proposed cut-off value to distinguish NSCLC patients from controls ranged from 2 to 25 ng/ml, with sensitivities values varying from 46 to 86% (81, 88, 91). Given this results, cfDNA concentration as a noninvasive strategy for early detection of LC was investigated among 1,035 heavy smokers monitored by annual CT for 5 years by Paci et al., but with disappointing results (87).

Human β-actin gene detected by RT-PCR was another frequent used method for cfDNA detection (83, 85, 86, 89, 90). While some studies failed in demonstrate the utility of cfDNA (83, 90), others have shown favorable results (85, 86, 89). Szpechcinski and colleagues studied not only LC patients and healthy controls, but also patients with benign lung diseases, found significantly higher plasma cfDNA levels among NSCLC patients than in those with chronic respiratory inflammation and healthy individuals (85, 86). A cut-off value of 2.8 ng/ml was proposed to discriminate NSCLC patients from healthy individuals, with sensitivity and specificity values ranging from 86 to 90% and 61 to 81%, respectively (85, 86).

The relationship between cfDNA levels and tumor histological type or staging is controversial, with several studies reporting no association (84, 86, 88, 90, 91), and others highlighting a difference related to disease staging (82, 83, 87).

#### cfDNA Genetic Alterations

##### Single Biomarker

Epidermal growth factor receptor (EGFR) is one of the most studied genes in LC, as the presence of certain mutations in this gene are considered markers of efficacy of target therapies. EGFR mutations detection in cfDNA has been also exploited in diagnostic setting (92–94, 96, 97), and two recent works focused on early stage LC patients. The first one, aimed to determine whether the electric field-induced release and measurement—EFIRM technology was able to detect exon 19 deletions and L858R EGFR mutations in patients with early stage NSCLC (97). The authors obtained a concordance rate between plasma and nodule biopsy of 100% and a global specificity of 95% (97). A second study, by Wan et al. (96) compared EGFR exon 19 deletions, T790M and L858R, using amplification-refractory-mutation system-based PCR assays (ARMS-PCR) in DNA isolated from nanoscale extracellular vesicles and cfDNA in NSCLC patients and controls. Although none of them were correlated with tumor volume, DNA isolated from extracellular vesicles was better than cfDNA for mutation detection among early stage NSCLC patients (96).

As TP53 is inactivated in most SCLC, Fernandez-Cuesta and colleagues (95) assessed the presence of exon 2 to 10 mutations in plasma cfDNA from 51 SCLC patients and 123 controls and showed that, despite their occurrence in control samples due to interference of somatic mutations, they were significantly more frequent in SCLC cases, even when stratified by stage (95).

##### Combination Biomarker

Despite recurrent point mutations in cancer-related genes, such as EGFR, have been frequently used, a non-negligible proportion of patients have no mutations in these selected genes. Instead of using only a single gene, several studies used multigene panels towards to improve the test performance. An early example is the CAncer Personalized Profiling by deep Sequencing (CAPP-Seq) developed by Newman and collaborators (98). This low-cost method covered multiple classes of somatic alterations and identified mutations in more than 95% of tumors, however showing low sensitivity for stage I patients (98). Nonetheless, in a final analysis, CAPP-Seq showed to potentially improve the low positive predictive value of LDCT screening (98). Cohen et al. (101) described the CancerSEEK, a blood test composed by levels of eight proteins and cfDNA mutations in 16 cancer-related genes that can detect eight frequent types of cancer, including LC. Globally, the results showed a sensitivity of 70% and a specificity of 99%. But a reduced sensitivity among stage I patients and a disappointing sensitivity for LC were noticed (101).

A malignancy prediction model for lung nodules was proposed by Ye et al. (102) in order to complement LDCT screening. Fixing the cut-off values in 4 for mutation score and in 0.3 for tumor mutation burden of cfDNA, the model predicted 33% of malignant adenocarcinoma samples with 100% specificity (102). In the same study, the concordance rate of driver mutations between cfDNA and tumor was low, suggesting that improving sensitivity of early stage LC detection by increasing sequencing depth or coverage may be inappropriate (102). More recently, a pilot investigation by Tailor et al. (104) using whole-exome sequencing (WES) in plasma cfDNA and matched peripheral blood mononuclear cell germline DNA from patients with a CT-detected pulmonary nodules, showed that the number of variants was significantly higher in the LC group than in controls and, when selecting 10 variants, 82% of LC patients were detected, showing the potential role for early LC detection in patients with CT-detected lung lesions (104).

#### cfDNA Epigenetic Alterations

##### Single-Dual Biomarker

One of the most studied epigenetic mechanisms is DNA methylation, which consists in the addition of a methyl group at the fifth carbon position of cytosine bases located 5′ to a guanosine in a CpG dinucleotide. Tumor suppressor gene hypermethylation results in gene silencing, occurs at early stages of cancer development, and is easily detected in cfDNA, mostly by methylation-specific PCR technologies (128).

The short stature homeobox 2 gene (SHOX2) is a known chondrocyte hypertrophy regulator, playing important functions in skeleton development, embryogenic pattern formation (129), embryonic morphogenesis, heart and nervous system development (130). SHOX2 methylation was investigated in respiratory (107, 110, 113, 115) and plasma samples (108, 113). When considered as a single biomarker, sensitivities for LC detection ranged from 68 to 89% in respiratory samples (107, 113) and from 60 to 81% in plasma (108, 113). Interestingly, SCLC histology presented the highest and stage I patients the lowest sensitivity values (107, 108). The performance of the *in vitro* diagnostic test kit Epi proLung BL Reflex Assay was assessed both in saccomanno-fixed bronchial and blood samples (110, 113). Analyzing SHOX2 and PTGER4 methylation in bronchial aspirates, Dietrich et al. (110) reported 78% sensitivity and 96% specificity in discriminating 125 LC cases from 125 controls. Interestingly, the sensitivity was higher in cytology positive samples, suggesting that this test may complement traditional investigations (110). Moreover, when respiratory and plasma samples were considered, the sensitivity increases, suggesting advantages in using a combined approach (113).

Methylation of other candidate genes was proposed for diagnostic biomarker in LC, including RASSF1A (106, 111, 116), HOXA9 (109, 116), Septin 9 (112) and DCLK1 (114). Ponomaryova et al. (111) showed that both RARB2, a tumor suppressor gene that encodes a retinoid acid nuclear receptor, and RASSF1A methylation were increased in stage I–III LC patients both in cfDNA and DNA bound to the blood cells surface. The best performance model reported included RARB2 and RASSF1A, both in plasma and bound to blood cells surface. Yet, the highest accuracy was found among stage III patients (111). SHOX2 (107) (108), HOXA9 (116), RASSF1A (116), and DCLK1 (114) hypermethylations seem to be more frequent among SCLC patients.

##### Combination Biomarkers

Combination of biomarkers seems to be a reasonable option to increase the performance of cfDNA methylation as a diagnostic marker. Nikolaidis et al. (121) suggested a set of four genes (TERT, WT1, p16 and RASSF1) to diagnose LC in bronchial lavage samples and, although sensitivity was improved in cytology-positive samples, the assay seems to be particularly useful in diagnosing cytology-negative LC. Interestingly, SCLC and squamous cell carcinomas were more detectable than adenocarcinomas (121). In a study by Ma and colleagues (123), using quantum dots-based (QDs-based) fluorescence resonance energy transfer (FRET) nanosensor technique to identify hypermethylation of a 3-gene panel, including PCDHGB6, HOXA9 and RASSF1, in bronchial brushings, a robust diagnostic performance for early-stage LC was reported, yet, sensitivity varied according to stage and histotype (123). The analysis of sputum samples of participants from the NELSON trial, demonstrated that, while sputum cytology did not detect any LC patients, a 3-gene panel, comprising RASSF1A, 3OST2 and PRDM14, detected 28% of cases 2 years before the diagnosis (126). Hulbert and colleagues (124) investigated subjects with suspicious nodules on CT imaging and built prediction models combining gene methylation with clinical information that correctly predicted LC in 91% of subjects using sputum and in 85% using plasma (124). From 20 tumor suppressor genes, Zhang et al. (119) found that nine (APC, CDH13, KLK10, DLEC1, RASSF1A, EFEMP1, SFRP1, RARb and p16INK4A) revealed a higher frequency of hypermethylation in stage I–II NSCLC than in cancer-free plasmas. Additionally, a 5-gene panel, comprising APC, RASSF1A, CDH13, KLK10 and DLEC1 achieved a sensitivity of 84% and specificity of 74% for early LC diagnosis (119).

More recently, Liang and collaborators created a plasma-based 9-marker diagnostic model to distinguish malignant from benign nodules, with a sensitivity of 80% and a specificity of 85%. The model was also very sensitive for early stages, which highlights its utility as complement to imaging methods (127). Interestingly, Ooki and colleagues (125), determined the clinical utility of a set of six genes, including CDO1, HOXA9, AJAP1, PTGDR, UNCX, and MARCH11, for predicting LC diagnosis not only in serum samples but also in pleural effusions and ascites. In serum, the panel reached a specificity of 71%, and a sensitivity of 72 and 60% for stage IA adenocarcinoma and squamous cell carcinoma, respectively. Promoter methylation of the six genes was significantly higher in cytology-positive pleural effusions and, when methylation of at least one of the four genes (CDO1, PTGDR, MARCH11, and UNCX) was considered, the sensitivity and specificity reached 70 and 85%, respectively. When AJAP1 was added to the panel, sensitivity increases and specificity drops, with similar findings for ascites, suggesting the utility of this gene panel for LC detection using different body fluids (125).

In conclusion, cfDNA concentration in plasma or serum samples seems to have diagnostic value in early-stages LC. As tumors-derived cfDNA is likely to represent the whole cancer genomic landscape, its genetic analysis has shown promising results. However, the genetic alteration or, more probably, a set of genetic changes with optimal diagnostic accuracy is still to be defined. Methylation is an early and frequently found epigenetic alteration that can be detected in cfDNA, not only from plasma or serum samples but also from respiratory specimens and other body fluids, representing an excellent opportunity for LC early diagnosis. Further studies are needed to find the optimal biomarker combination. For example, a single tube liquid biopsy allowing simultaneous analysis of cfDNA, tumor-derived extracellular vesicles and CTC with high and low EpCAM expression proved to be useful in predicting survival among advanced NSCLC (131). In the future, a similar combination biomarker strategy may be employed in diagnostic setting.

### Circulating Tumor Cells

A meta-analysis demonstrated that CTCs detection seems to be associated to lymph nodal metastasis and staging but not to histology (132). Since CTCs are very rare in bloodstream and are surrounded by normal peripheral blood cells, such as mononuclear and red blood cells (33), several techniques have been developed to selectively enrich CTCs and remove other blood cell components. These assays are classified as label-dependent, which includes EpCAM-based technologies (positive selection) and depletion of CD45-positive leukocytes (negative selection), and label-independent approaches, in which CTCs are separated based on CTCs physical or biological properties. The combination of these approaches may be used. After enrichment, CTCs need to be characterized, usually through the identification of tumor-associated proteins, mRNA or DNA, using several strategies that includes fluorescence immunocytochemistry, RT-PCR, next-generation sequencing (NGS) and whole-genome amplification (33, 133).

Several studies evaluated the utility of CTCs in diagnosing LC (**Table 5**). The CellSearch system, an EpCAM-based technology approved by FDA, has been investigated in LC diagnosis. Allard and collaborators studied a population of healthy subjects, non-malignant diseases, and patients with a variety of metastatic carcinomas, including LC. A cut-off of ≥2 CTCs/7.5 ml blood only identified 20% of LC patients (134). A prospective study showed that CTCs were detected in 30.6% of LC and in 12.0% of non-malignant disease patients and, despite CTC count was significantly higher among the first group, had a low discriminatory capacity (135). However, metastatic and non-metastatic LC patients were successfully distinguished (135).

**Table 5:** Circulating tumor cells (CTC) as biomarker for lung cancer diagnosis.

Study	Year	Assay	Study population		Diagnostic performance		
				Cut-off	S	E	AUC
Allard (134)	2005	CellSearch system	145 healthy women 199 women with non-malignant disease 964 metastatic cancer patients including 99 LC	≥2 CTCs/7.5 ml	20%	99%	–
Tanaka (135)	2009	CellSearch system	25 patients with non-malignant lung disease 125 LC patients (22 SCC, 85 ADC, 9 SCLC, 9 other) Stages: I-III: 94; IV: 31	≥1 CTCs/7.5 ml	30%	88%	0.598
Hofman (136)	2011	ISET method	39 healthy subjects 208 NSCLC (54 SCC, 115 ADC, 39 others) Stages: I: 86; II: 51; III: 58; IV: 13	≥1 CTCs/ml	37%	100%	–
Hofman (137)	2011	CellSearch system vs. ISET method	40 healthy subjects 210 NSCLC (57 SCC, 120 ADC, 33 other) Stages: I: 91; II: 40; III: 60; IV: 19	≥1 CTCs/ml	ISET: 50%CellSearch: 39%Combined: 69%	100%	
Hofman (138)	2012	ISET method	59 healthy subjects 250 NSCLC patients (67 SCC, 150 ADC, 33 others) Stages: I: 111; II: 70; III: 50; IV: 19	≥1 CTCs/ml	41%	100%	–
Ilie (139)	2014	ISET method	77 non-COPD controls (42 smokers and 35 healthy non-smoking individuals) 168 COPD patients	≥1 CTCs/ml	All COPD patients in which CTCs were found (5%) developed LC during follow-up.	Non-COPD controls: 100%COPD controls: 95%	–
Dorsey (140)	2015	Telomerase-promoter immunofluorescence-based assay	Healthy controls 30 NSCLC patients referred for definite RT	≥1 CTCs/ml	65%	100%	–
Fiorelli (141)	2015	Isolation by size method	77 patients with a single lung lesion: 17 benign lesions and 60 with LC (29 ADC, 18 SCC, 13 LCC Stages: I: 25; II: 19; III: 10; IV: 6	>25 CTCs/ml	89%	100%	0.900
Chen (142)	2015	Ligand-targeted PCR for folate receptors	56 healthy volunteers 227 patients with benign lung disease 473 NSCLC patients (293 ADC, 103 SCC, 77 others) Stages I: 18; II: 5; III: 127; IV: 323	≥1 CTCs/3ml	76%	82%	0.813
Xu (143)	2017	Negative enrichment using anti-CD45 coated magnetic beads and CD45 depletion cocktail vs unbiased method	151 non-cancerous controls 83 LC patients Stages: I: 13; II: 10; III: 33; IV: 27	≥1 CTCs/ml	Anti‐CD45 coated magnetic beads group: 62%CD45 depletion cocktail group: 47%Unbiased group: 92%	94%	–
Xue (144)	2018	Ligand-targeted PCR for folate receptors	24 patients with benign lung diseases and 2 healthy subjects 72 LC patients (50 ADC, 14 SCC, 8 others) Stages: 0-IS: 2; I: 31; II: 7; III: 12; IV: 14; Uncertain: 6	8.7 CTC/3 mL	82%	73%	0.822
Frick (145)	2020	Telomerase-promoter immunofluorescence-based assay	92 NSCLC undergoing SBRT (22 ADC, 15 SCC, 55 not confirmed) Stages: IA: 81; IB: 11	≥1 CTCs/ml	41%	–	–
He (146)	2017	GILUPI CellCollector *in vivo*	19 healthy volunteers 32 ground-glass nodules patients 15 advanced LC patients	≥1 CTCs/7.5ml	GGN group: 16%Advanced LC patients: 73%	100%	–
Duan (147)	2020	GILUPI CellCollector *in vivo*	20 healthy subjects 44 suspected LC patients including 10 patients diagnosed with benign lung diseases and 34 LC (all ADC)Stages 0: 11/34; IA: 23/34	≥1 CTC/ml	53%	Healthy controls: 100%Benign lung disease: 90%	Benign lung disease: 0.715
He (148)	2020	GILUPI CellCollector *in vivo*	72 matched healthy controls 24 LC patients (6 SCC and 18 ADC) Stages: I: 18/24; II: 6/24	≥1 CTC/ml	63%	100%	–

ADC, adenocarcinoma; AUC, area under the curve; COPD, chronic obstructive pulmonary disease; E, specificity; IS, in situ; ISET, solation by size of epithelial tumor cell; GGN, ground-glass nodules; LC, lung cancer; NSCLC, non-small cell lung cancer; S, sensitivity; SCC, squamous cell carcinoma; SCLC, small cell lung cancer.

Isolation by size of epithelial tumor cell (ISET) has been investigated in LC early diagnosis. In 2011, Hofman et al. (136) reported a mean of 42 circulating nonhematologic cells detected in 49% of NSCLC patients undergoing surgery, 37% with malignant features and no cells were found in the control healthy group (136). One year later, the same authors achieved similar conclusions in a larger population (138). Ilie and colleagues (139) examined the presence of CTCs in complement to CT-scan in patients with chronic obstructive pulmonary disease (COPD) in order to identify early LC. CTCs were detected in 5% of COPD patients and all of them developed LC after a mean follow-up period of 3.2 years, suggesting that monitoring CTC-positive COPD patients may allow early LC diagnosis. Importantly, a study comparing CellSearch and ISET methods in LC diagnosis showed that CTCs can be detected by both methods. Moreover, they may complement each other since the percentage of patients with detected CTCs is higher when combining the two methods with a higher number of CTCs detected by ISET (137). Obstacles to CellSearch method include epithelial–mesenchymal transition phenomena and epithelial nontumor cells in circulation (133, 149). Even though CTCs detected by CellSearch are able to predict the prognosis among NSCLC patients reflecting their clinical relevance (150, 151).

Anti−cluster of differentiation, CD45 antibody−coated magnetic beads, have been used for leucocyte depletion and negatively enrich CTCs. Although this method has a high sensitivity (152), CTCs and leucocytes may aggregate and form clusters, or even CTCs may be loss in the process (153). Xu et al. (143) compared this negative enrichment method to an unbiased detection method, in which erythrocytes were lysed and removed and the remaining nucleated cells were bound to substrates, fixed, stained using fluorescence−labeled antibodies and, thereafter examined by microscopy. The results demonstrated that unbiased detection method efficiently detected 92.2% of CTCs among LC patients, and 65% of early-stage LC patients. By contrast, only 40–60% of CTCs were detected by negative enrichment (143). These results suggest that unbiased detection methods may detect CTCs in early-stage LC patients, also revealing a better sensitivity than negative enrichment methods.

More recently, the diagnostic value of detecting folate receptors (FR)-positive CTC by a novel ligand-targeted polymerase chain reaction method in NSCLC patients was investigated (154, 155). Chen and collaborators (142) showed that CTC levels in NSCLC patients were significantly higher than in those with benign lung diseases and in healthy donors. Also, CTC detection was able to identify NSCLC patients (AUC = 0.813). Moreover, a joint model combining CTC, carcinoembryonic antigen, neuron-specific enolase, and Cyfra21-1 was efficient in NSCLC diagnosis (142), showing the interest of combining different types of biomarkers. Later, Xue et al. (142) reported high sensitivity and specificity values using a cut-off value of 8.7 CTC Units/3 ml in discriminating early-stage LC patients from controls (142).

A telomerase-promoter-based assay has shown to be able to overcome the current limitations in detecting CTCs. In a pilot study with 30 patients referred for definite radiotherapy (RT), Dorsay et al. (140) showed a successful detection of CTCs in 65% of patients, being the median CTC counts in patients before RT significantly higher than post-RT values. Interestingly, one patient was exception and developed metastatic disease soon after RT (140). Also in RT setting, 41% of early-stage NSCLC patients had a positive CTC test prior to treatment (145).

Other recent approach is the GILUPI CellCollector *in vivo* examination technique, which consists in a structured and functionalized medical wire that captures CTCs directly from the bloodstream and identifies them through the cytokeratin immunofluorescence intensity signal (156). He and collaborators demonstrated that this strategy was able to identify not only 73.3% of the ad.vanced LC patients as reported before (157), but also 15.6% of the ground-glass nodules (146). In two later studies including early LC patients, sensitivity values of 53–63% were reached (147, 148). Remarkably, the captured CTCs can be separated for NGS or PD-L1 analysis (146–148).

Evidence has shown that CTCs are useful as biomarkers in LC diagnosis. However, techniques for CTCs isolation and counting still need to be optimized and harmonized so that can be possible to validate the ideal detection method. New techniques, such as microfluidic technologies have shown exciting results (158). In order to assess technical validity of emerging CTC detection methods and generate comparative data, a platform was recently created to help to define minimal requirements for performance qualification prior to clinical validation (159).

### MicroRNAs

Since miRNA fragments are stable in blood and evidence suggests that their landscape in peripheral circulation correlate with the original tumor (160), they represent a valuable potential biomarker for LC diagnosis.

#### Serum and Plasma Samples

Notable performances in discriminating between LC patients and healthy and/or benign lesions controls were reported by several authors (**Table 6**.). However, most studies included metastatic and locally advanced patients, and only some of them were restricted to early stages (168, 170, 178, 185, 188).

**Table 6:** MicroRNA (MiRNA) as biomarker for lung cancer diagnosis.

Study	Year	Assay	Tested miRNA	Study population	Sample	Diagnostic performance			
						Best predictors	S	E	AUC
Chen et al. (161)	2008	qRT-PCR and Solexa sequencing	63 miRNAs	75 healthy individuals 152 LC patients	Serum	miR-25, miR-223	–	–	–
Xie et al. (162)	2009	RT-PCR	miR-21 and miR-155	17 healthy individuals 23 NSCLC patients (13 ADC and 10 SCC) Staging: I: 3; II: 5; III: 7; IV: 8	Sputum	miR-21	70%	100%	0.902
Yu et al. (163)	2010	RT-PCR	7 miRNAs (miR-486, miR-126, miR-145, miR-21, miR-182, miR-375, and miR-200b)	Discovery set: 20 stage I ADC Case-control set: 36 healthy individuals 36 stage I ADC Validation set: 58 healthy individuals 64 NSCLC patients (33 ADC and 31 SCC) Validation set staging: I: 16; II: 15; III: 17; IV: 16	Sputum	miR-21, miR-486, miR-375, miR-200b	70%	80%	0.839
Shen et al. (164)	2011	RT-PCR	12 miRNAs (miR-21, 126, 145, 139, 182, 200b, 205, 210, 375, 429, 486-5p, and 708)	29 healthy individuals 58 NSCLC patients including 34 ADC and 24 SCC Staging: I: 15; II: 15; III: 12; IV: 16	Plasma	miRNA-21, -126, -210, and 486-5p	86%	97%	0.926
Shen et al. (165)	2011	RT-PCR	5 miRNAs (miR-21, miR126, miR210, miR375, miR-486-5p)	- 80 benign SPNs patients - 76 malignant SPNs patients including 40 adenocarcinomas and 36 squamous cell carcinomas Staging: I: 24; II: 30; III–IV: 22	Plasma	miR-21, miR- 210, and miR-486-5p	76%	85%	0.855
Zheng et al. (166)	2011	RT-PCR	15 miRNAs (miR-17, -21, -24, -106a, -125b, -128, -155, -182, -183, -197, -199b, -203, -205, -210 and -221)	68 healthy individuals 74 LC patients: -17 SCLC -48 NSCLC (18 ADC, 23 SCC, 7 LCC and 9 others (carcinoid or mixed tumor) Staging: I: 21; II: 12; III: 11; IV: 30	Plasma	miR-155, miR-197, miR-182	81%	87%	0.901
Boeri et al. (167)	2011	TaqMan Microfluidic cards	15 miRNAs	81 heavy smokers’ controls 53 LC patients (including 30 ADC) Staging: I: 28; II–III–IV: 25	Plasma	miR-17, miR-660, miR-92a, miR-106a, and miR-19b	75%	100%	0.880
Foss et al. (168)	2011	RT-PCR	11 miRNAs (miR-1268, miR-574-5p, miR-1254, miR-1228, miR-297, miR-1225-5p, miR-923, miR-1275, miR-185, miR-483-5p, miR-320a)	Discovery set: 11 healthy individuals; 11 early-stage (I and II) NSCLC patients Validation set: 31 healthy individuals; 22 early-stage (I and II) NSCLC patients	Serum	miR-1254 and miR-574-5p	73%	71%	0.750
Bianchi et al. (169)	2011	RT-PCR	34 miRNAs	30 healthy individuals 22 ADC 12 SCC Staging: I: 22; II–IV: 12	Serum	–	71%	90%	0.890
Heegaard et al. (170)	2012	RT-PCR	30 miRNAs	220 early-stage NSCLC patients 220 healthy individuals Staging: I: 180; II: 40	Serum	miR-146b, miR-221, let-7a, miR-155, miR-17-5p, miR27a, miR-106a, miR-29c	–	–	0.602
Sozzi et al. (171)	2014	RT-PCR	24 miRNAs	870 healthy individuals (690 smokers) 69 LC patients (55 smokers) Staging: I: 37; II–III: 12; IV: 19	Plasma	–	87%	81%	–
Shen et al. (172)	2014	RT-PCR	12 miRNAs (miRs-21, 31, 126, 139, 182, 200b, 205, 210, 375, 429, 486, and 708)	Training set: 68 cancer-free smokers; 66 LC patients (27 ADC, 26 SCC and 13 SCLC) Training set staging of the NSCLC patients: I: 17; II: 18; III–IV: 18 Testing set: 73 cancer-free smokers; 64 LC patients (30 ADC, 28 SCC, 6 SCLC) Testing set staging of the NSCLC patients: I: 19; II: 19; III–IV: 20	Sputum	miR-31, miR-210	65%	89%	0.830
Wang et al. (173)	2014	RT-PCR	9 miRNAs(miR-20a, miR-25, miR-486-5p, miR-126, miR-125a-5p, miR-205, miR-200b, miR-21, and miR-155)	111 healthy individuals 142 LC patients (including 101 ADC, 22 SCC and 10 SCLC) Staging: I: 70; II: 24; III: 21; IV: 27	Serum	miR-125a-5p, miR-25, and miR-126	88%	83%	0.930
Montani et al. (174)	2015	RT-PCR	34 miRNAs	972 healthy individuals 36 LC patients (28 ADC and 5 SCC) Staging: I: 31; II–III: 5	Serum	miR-92a-3p, miR-30b-5p, miR-191-5p, miR-484, miR-328-3p, miR-30c-5p, miR-374a-5p, let-7d-5p, miR-331-3p, miR-29a-3p, miR-148a-3p, miR-223-3p, miR-140-5p	75%	78%	0.850
Xing et al. (175)	2015	RT-PCR	13 miRNAs (miR205; miR708; miR375; miR200b; miR182; miR155; miR372; miR143; miR486-5p; miR126; miR31; miR21; miR210)	Training set: 62 benign SPNs; 60 malignant SPNs (with 27 ADC and 29 SCC) Training set staging: I: 39; II: 21 Internal Testing set: 69 benign SPNs; 67 malignant SPNs (including 30 ADC and 31 SCC) Internal Testing set staging: I: 45; II: 22 External Testing set: 79 benign SPNs; 76 malignant SPNs (including 34 ADC and 35 SCC) External Testing set staging: I: 51; II: 25	Sputum	miR-21, miR-31, miR-210	82–88%	81–87%	0.920
Wang et al. (176)	2015	RT-PCR	16 miRNAs (miR-193a-3p, miR-214, miR-7, miR-25, miR-483-5p, miR-523, miR-885-5p, miR-520c-3p, miR-484, miR-720, miR-133a, miR-337-5p, miR-150, miR-1274b, miR-342-3p, miR-145)	48 healthy individuals 56 benign nodules 108 NSCLC patients (including 52 ADC and 27 SCC) Staging: I: 43; II: 15; III: 29; IV: 17	Serum	miR-483-5p, miR-193a-3p, miR-214, miR-25, and miR-7	95%	84%	0.952
Kim et al. (177)	2015	RT-PCR	5 miRNAs (miR-21, miR-143, miR-155, miR-210, and miR-372)	10 cancer-free controls 21 early-stage NSCLC patients (13 ADC, 5 SCC and 3 LCC) Staging: I: 12; II: 9	BAL fluid/Sputum	miR-21, miR-143, miR-155, miR-210, and miR-372	Patients BAL vs controls sputum: 86%Sputum: 68%	Patients BAL vs controls sputum: 100%Sputum: 90%	
Li et al. (178)	2015	RT-PCR	10 miRNAs (miR-126, miR-150, miR-155, miR-205, miR-21, miR-210, miR-26b, miR-34a, miR-451 and miR-486)	11 healthy individuals 11 early-stage NSCLC patients Staging: I–II: 11	Plasma	miR-486 and miR-150(individually)	miR-486: 91%miR-150: 82%	miR-486: 82%miR-150: 82%	miR-486: 0.926miR-150: 0.752
Fan et al. (179)	2016	Fluorescence quantum dots liquid bead	12 miRNAs (miR-15b-5p, miR-16-5p, miR-17b-5p, miR-19-3p, miR-20a-5p, miR-28- 3p, miR-92-3p, miR-106-5p, miR-146-3p, miR-506, miR-579, and miR-664	54 healthy individuals 70 NSCLC patients (56 ADC, 12 SCC and 2 LCC) Staging: I: 49; II–III: 21	Serum	miR-15b-5p, miR-16-5p, miR-20a-5p	94$	94%	0.930
Razzak et al. (180)	2016	RT-PCR	3 miRNAs (miR-21, miR-210, miR-372)	10 healthy individuals 21 Early-stage NSCLC patients (including 13 ADC and 4 SCC) 22 Advanced-stage NSCLC patients (10 ADC and 5 SCC) Staging: I: 14; II: 7; III: 14; IV: 8	Sputum	miR-21, miR-210, miR-372	67%	90%	0.926
Bagheri et al. (181)	2017	RT-PCR	6 miRNAs (miR-223, miR-212, miR-192, miR-3074, SNORD33 and SNORD37)	17 healthy individuals 17 NSCLC patients (11 ADC and 6 SCC) Staging: I: 2; II: 3; III: 5; IV: 7	Sputum	miR-223	82%	95%	0.900
Leng et al. (182)	2017	RT-PCR	54 miRNAs	30 cancer-free smokers 34 NSCLC patients (21 AC and 13 SCC) Staging: I: 19; II: 9; III–IV: 15	Plasma	miRs-126, 145, 210, and 205-5p	92%	97%	0.960
Lu et al. (183)	2018	RT-PCR	13 miRNAs (miR-101, miR-133a, miR-17,miR-190b,miR-19a, miR-19b, miR-205, miR-26b, miR-375, miR-451, miR-601, miR-760, miR-765)	203 normal individuals 258 LC patients (133 ADC, 76 SCC and 49 SCLC) Staging: I: 78; II: 27; III: 40; IV: 64	Plasma	miR-17, miR-190b, miR-19a, miR-19b, miR-26b, and miR-375	80%	80%	0.868
Abu-Duhier et al. (184)	2018	Magnetic bead technology and TaqMan assays	miRNA-21	80 healthy individuals 80 NSCLC patients (60 ADC and 20 SCC) Staging: I: 2; II: 7; III: 26; IV: 46	Plasma	–	80%	80%	0.891
Xi et al. (185)	2018	RT-PCR	12 miRNAs (miRNA-17, -146a, -200b, -182, -155, -221, -205, -126, -7, -21, -145, and miRNA-210)	15 benign pulmonary nodules 42 NSCLC patients Staging: IA: 29; IB: 10; II: 3	Plasma	miRNA-17, -146a, -200b, -182, -221, -205, -7, -21, -145, -210 (individually)	>55%	>60%	>0.680
Li et al. (186)	2019	RT-PCR	4 miRNAs (miRs-126-3p, 145, 210-3p and 205-5p)	245 cancer-free smokers 239 NSCLC cases (111 AC, 102 SCC and 26 LCC) Staging: I: 72; II: 76; III–IV: 91	Plasma	miRs-126-3p, 145, 210-3p and 205-5p	90%	95%	–
Liang et al. (187)	2019	RT-PCR	miRNA-30a-5p	20 healthy individuals 38 lung benign lesions104 LC patients (including 75 ADC, 20 SCC and 5 SCLC) Staging: I–IIA: 62; IIB–IV: 42	Plasma	–	80%	61%	0.820
Xi et al. (188)	2019	RT-PCR	10 miRNAs (miR-17, -146a, -200b, -182, -221, -205, -7, -21, -145, and miR-210)	13 benign pulmonary nodules 39 NSCLC patients staging: 0–IA: 31; IB: 7; IIA: 1	Plasma	miRNA-146a, -200b, and -7	72%	69%	0.781
Liao et al. (189)	2020	RT-PCR	2 miRNAs in sputum (miRs‐31‐5p and 210‐3p)3 miRNAs in plasma (miRs‐21‐5p, 210‐3p, and 486‐5p)	55 cancer-free smokers 56 NSCLC patients (31 ADC and 25 SCC) Staging: I: 18; II: 17; III–IV: 21	Plasma and Sputum	Sputum: miRs‐31‐5p and 210‐3pPlasma: miRs‐21‐5p	84%	91%	0.930

ADC, adenocarcinoma; AUC, area under the curve; BAL, bronchoalveolar lavage; E, specificity; LC, lung cancer; NSCLC, non-small cell lung cancer; RT-PCR, real time polymerase chain reaction; S, sensitivity; SCC, squamous cell carcinoma; SCLC, small cell lung cancer; SPN, solitary pulmonary nodules.

Foss and collaborators demonstrated that miR-1254 and miR-574-5p were significantly increased in serum samples from early-stage NSCLC, achieving a sensitivity and specificity of 73 and 71%, respectively, in differentiating from controls (168). Fan et al. (179) evaluated the miRNA expression in serum samples of NSCLC patients and healthy subjects firstly by qRT-PCR and, thereafter, they validated the results using the fluorescence quantum dots liquid bead array. They found that five miRNAs including miR-16-5p, miR-17b-5p, miR- 19-3p, miR-20a-5p, and miR-92-3p were significantly downregulated, while miR-15b-5p was upregulated among NSCLC patients. A 3-miRNA profile (miR-15b-5p, miR-16-5p, miR-20a-5p) using bead array showed to be the best diagnostic approach with high sensitivity and specificity values (179). Both serum and plasma samples from 220 early stage NSCLC patients and 220 matched controls were studied by Heegaard et al. (170) who reported that remarkably the expression levels in serum did not correlate with those in plasma, and while in serum samples from NSCLC patients a decreased expression of miR-146b, miR-221, let-7a, miR-155, miR-17-5p, miR-27a and miR-106a and an increased expression of miR-29c were noticed, no significant differences were stated on miRNAs plasma levels.

More recently, the values of plasma miR-486 and miR-150 for LC early diagnosis were also studied by Li et al. (178). The authors found that, individually, these miRNAs were able to distinguish LC patients from healthy volunteers with reported sensitivity and specificity higher than 80% (178). A single-center study reported significantly higher levels of miRNA-17, -146a, -200b, -182, -221, -205, -7, -21, -145, and miRNA-210 in NSCLC nodules comparing with benign ones (185). Later, the same group built a prediction model including 3 miRNAs (miRNA-146a, -200b, and -7) and CT features such as pleural indentation and spiculation, with high diagnostic value in early-stage NSCLC (188).

Circulating miRNAs value in LC screening programs has been widely investigated with promising results. A study by Boeri et al. (167), including participants from two CT-based screening cohorts, INT/IEO and MILD, explored miRNA expression profiles in plasma samples collected from patients 1 and 2 years before CT-detected lesions compared with a control group of heavy-smoking individuals. A signature of 15 miRNAs could discriminate both groups with a sensitivity 80%, and, a specificity of 80 and 90%, respectively (167). Notably, the predictive value of this signature was evaluated to be useful up to 28 months before the disease, with mir-660, mir-140-5p, mir 451, mir-28-3p, mir-30c, and mir-92a being the most frequently deregulated miRNAs (167). The most frequently miRNA deregulated at the time of LC diagnosis were mir-17, mir-660, mir-92a, mir-106a, and mir-19b (167). Later, the same research group analyzed the plasma samples from 939 participants, including 69 LC patients and 870 disease-free individuals in two arms (LDCT and observation) and using a miRNA signature classifier comprising 24 miRNAs reported a diagnostic performance for LC detection of 87% for sensitivity and 81% for specificity for both arms, and a negative predictive value of 99% (171). Furthermore, when combined with LDCT, a significant reduction of LDCT false positives was noticed (171). The BioMILD trial (190) consists in a LC screening program combining LDCT and circulating miRNA which prospectively enrolled 4,119 volunteers from a single center. Preliminary analysis presented in IASLC by Pastorino and colleagues showed a higher LC incidence and overall mortality in subjects with positive LDCT and/or miRNA at baseline and no detrimental effects on stage I LC proportion, resection rates, or interval cancer incidence in the group of subjects that completed 3-year LDCT repetition, suggesting that the combination of these tools is a valuable, safe and reduce unnecessary LDCT repeats (191). After Bianchi et al. (169) developed a serum 34 miRNAs panel able to identify patients with NSCLC in an asymptomatic high-risk population. Montani and colleagues (174) performed a multicenter study enrolling 1,115 high-risk individuals from the Continuous Observation of Smoking Subjects (COSMOS) LC screening program and reduced the original serum 34-miRNA signature to 13 miRNAs (miR-92a-3p, miR-30b-5p, miR-191-5p, miR-484, miR-328-3p, miR-30c-5p, miR-374a-5p, let-7d-5p, miR-331-3p, miR-29a-3p, miR-148a-3p, miR-223-3p, miR-140-5p), maintaining the same performance (174).

A systematic review and metanalysis published in 2017 comprising a total of 134 studies, with 6,919 LC patients and 7,064 controls, confirmed the good diagnostic performance of miRNA (192). Moreover, a subgroup analysis showed that combining miRNAs and Caucasian populations yield higher diagnostic performances, serum might serve as an ideal sample type and that the diagnostic role of miRNAs in early stage LC was high (192). Besides, some miRNAs, such as miR-21-5p, miR-223-3p, miR-155-5p and miR-126-3p, were pointed out as potential biomarkers (192). A more recent review confirmed the high diagnostic performance of miRNA in early detection of LC and also highlighted that multiple miRNA-based panels generally performed better than individual markers (193).

#### Respiratory Samples

Sputum is the most easily accessible biological fluid and its cytological analysis has been used for LC diagnosis despite its low sensitivity. Molecular analysis of sputum might be more sensitive than cytology (194, 195). Several studies assessed the role of sputum in diagnosis of LC (162, 163, 172, 175, 180, 181, 196).

For example, a panel of three sputum miRNAs (*miRs-21*, *31*, and *210*) allowed to differentiate between malignant and benign nodules, with sensitivity and specificity values higher than 80% (175). Additionally, other specifications were explored: overexpression of *miR-21* was associated with adenocarcinoma, whereas *miR-210* was related to squamous cell carcinoma, and the expression level of *miR-31* associated with smoking. These findings suggested that sputum miRNA biomarkers may improve LC screening in heavy smokers (175). Also, the same research group showed that a panel of four miRNAs (*miR-21*, *miR-486*, *miR-375*, and *miR-200b*) could distinguished LC patients from controls with high sensitivities and specificity values, without differences among the stage subgroups, with the best prediction for adenocarcinoma (196).

Indeed, CT scan has an important role in LC diagnosis, however with low specificity. Sheng et al. (172) determine whether analysis of the miRNA signatures could improve regular CT scan and concluded that a panel of two miRNAs could cover the major histological types. Taken together, the combination of miRNA biomarkers and CT provided a higher specificity than CT alone (172).

Showing that respiratory samples other than sputum may have value, Kim and collaborators (177) investigated the role of 5 miRNAs (miR-21, miR-143, miR-155, miR-210, and miR-372) in discriminate early NSCLC patients from controls using both sputum and BAL samples and reported better diagnostic performance with BAL (177). Very recently, Liao et al. (189) determined a higher expression level of two sputum miRNAs (*miRs-31-5p* and *210-3p*) and three plasma miRNAs (*miRs-21-5p*, *210-3p*, and *486-5p*) of 76 NSCLC patients and 72 cancer-free smokers. Considered these panels, the authors reported 65.8–75.0% sensitivities and 83.3–87.5% specificities for LC diagnosis (189). Moreover, the expression levels of both *miR-21-5p* and *miR-210-3p* in sputum was correlated to squamous cell carcinoma (189). These results suggest that the combination of markers from different body fluids may play a role.

Overall, it seems that some miRNAs, either in circulation or in respiratory samples, have a role as biomarkers for early cancer diagnosis, especially when used in combination and as a complement to LDCT. However, the current data consist mostly in small sized populations from single-center studies with a great variability in terms of staging, analyzed miRNA and methodologies. Despite the great potential of miRNA, larger validation studies are required in order to define their exact role in clinics.

### Exosomes

Exosomes were found to be increased in LC patients compared with healthy controls (197). Several techniques have been used for exosome isolation, including methods based on physical features, such as ultracentrifugation, density gradient separation, ultrafiltration, size exclusion chromatography, chemical precipitation methods, and biological assays such as immune-bead isolation. Transmission electron microscopy and western blot are two examples of frequently used techniques for further exosome characterization. Additionally, commercial kits are available (54).

Exosomal miR-21 is a potential biomarker for cancer diagnosis, including LC. However, may be increased in other types of cancer, as well as in other diseases which suggests that the combination of miRNA panels may provide better results (24). In an early study, Cazzoli et al. (198) reported that a panel of six exosomal miRNAs (miR-151a-5p, miR-30a-3p, miR-200b-5p, miR-629, miR-100 and miR-154-3p) were able to discriminate LC from granuloma patients, with a sensitivity and specificity of 96 and 60%, respectively (AUC = 0.760) (198). Another 6-miRNA panel comprising miR-19b3p, miR-21-5p, miR-221-3p, miR-409-3p, miR-425-5p and miR-584-5p was able to discriminate lung adenocarcinoma patients from healthy controls, achieving an AUC of 0.84 (199). Likewise, Jin et al. (200) selected a panel of exosomal miRNAs (let-7b-5p, let-7e-5p, miR-23a-3p and miR-486-5p) and obtained a sensitivity of 80% and a specificity of 92% (AUC = 0.899), regarding ability to differentiate early-stage NSCLC from non-NSCLC patients. Moreover, adenocarcinoma and squamous cell carcinoma histology was identified by combining miR-181b-5p with miR-361b-5p (AUC = 0.936), and miR-10b-5p with miR-320b (AUC = 0.911), respectively (200). Also exosomes might be useful in identified malignant pleural effusions. Lin et al. (201) demonstrated a higher expression of miR-205p5p and miR-200b in pleural effusions of LC patients comparing with those with infections (201). Exosomes may be also useful in identifying tumor somatic mutations, such as EGFR activating mutations (202, 203). In addition, some studies have explored the potential of exosomal proteins as diagnostic biomarkers (204–207). More recently, Zhang and colleagues (208) demonstrated that a four biomarker panel, including miR-17-5p, carcinoembryonic antigen (CEA), cytokeratin 19 fragment (CYFRA21-1), and squamous cell carcinoma antigen (SCCA), was able to reach an adequate diagnostic performance (AUC = 0.844) (208).

These data suggest that exosomes, specially exosomal miRNAs, due to its stability, may represent valuable diagnostic biomarkers achieving promising sensitivity and specificity results. Although some technical concerns have been raised (209), the technological advances have the potential to overcome these difficulties and to allow the development of more robust assays to be part of the routine clinical practice in the future.

### Tumor-Educated Platelets

Evidence have shown that platelet RNA profile is distinct in patients with and without cancer, the later expressing a highly dynamic mRNA repertoire both at cancer onset and progression as well as during the treatment (25, 210). Best et al. (210) prospectively characterized the platelet mRNA profiles in 55 healthy donors and 228 patients with localized and metastasized tumors and concluded that cancer patients could be discriminated from non-cancer individuals with 97% sensitivity, 94% specificity and 96% accuracy (AUC = 0.986). In a multiclass analysis, the authors further distinguished healthy donors from patients with specific types of cancer with an average accuracy of 71% (210). Biomarkers like KRAS, EGFR, MET, HER2 or PIK3CA were also accurately distinguished using surrogate TEP mRNA profiles (210) as well as EML4-ALK rearrangements (211).Other studies have also shown that the analysis of mRNA profiles may allow to detect the primary tumor (212–214). Also, susceptibility to metastasis and staging seems to be possible to predict (25). Calverley et al. (215) identified a subset of platelet-based gene expression that are differentially expressed in individuals with LC metastases (215).

TEPs may have advantages over other blood-based biosources: they are abundant, may be easily isolated and acquire specific RNA from tumor cells. Since platelets have a median of 7 days of survival, expression of a highly dynamic mRNA repertoire is expected during cancer progression. However, more robust and specific studies are needed to better define the value of TEPs applicability in LC early detection, alone or in combination with other blood-based biomarkers or diagnostic procedures.

## Future Perspectives

While liquid biopsy-derived biomarkers have shown promising results in the early detection of LC, currently, there is no evidence for its use in the screening or diagnosis of LC outside the research setting. These biomarkers are still limited by a significant proportion of false negatives and a negative plasma test still requires a confirmatory tissue biopsy. Indeed, low or absent values of most liquid biopsy components in the very early stages of LC limit their applicability. Due to tumour heterogeneity, to the emergence of different tumours or pre-cancerous conditions and using conventional tissue biopsy as a reference, positive results not related with LC can also be expected, and their clinical significance needs further clarification. The technological advances expected to happen in the next few years may be able to help mitigate these limitations, with the development of more sensitive and specific assays. Biomarker combination and the combined use of biomarkers and other diagnostic tools, such as imaging techniques, seem to be a promising strategy, although the best combinations are still to be defined. Larger and more robust studies are required to define and validate the role of liquid biopsy in the mainstream clinical practice for the screening or diagnosis of LC.

## Author Contributions

All authors listed have made a substantial, direct, and intellectual contribution to the work and approved it for publication.

## Funding

This work is financed by the ERDF—European Regional Development Fund through the Operational Programme for Competitiveness and Internationalization—COMPETE 2020 Programme and by National Funds through the Portuguese funding agency, FCT—Fundação para a Ciência e a Tecnologia within project POCI-01-0145-FEDER-030263.

## Conflict of Interest

The authors declare that the research was conducted in the absence of any commercial or financial relationships that could be construed as a potential conflict of interest.
